# Tumor Extracellular Matrix Remodeling: New Perspectives as a Circulating Tool in the Diagnosis and Prognosis of Solid Tumors

**DOI:** 10.3390/cells8020081

**Published:** 2019-01-23

**Authors:** Marta Giussani, Tiziana Triulzi, Gabriella Sozzi, Elda Tagliabue

**Affiliations:** 1 Molecular Targeting Unit, Department of Research, Fondazione IRCCS Istituto Nazionale dei Tumori di, 20133 Milano, Italy; marta.giussani@istitutotumori.mi.it (M.G.); tiziana.triulzi@istitutotumori.mi.it (T.T.); 2 Tumor Genomics Unit, Department of Research, Fondazione IRCCS Istituto Nazionale dei Tumori di, 20133 Milano, Italy; gabriella.sozzi@istitutotumori.mi.it

**Keywords:** extracellular matrix, circulating biomarkers, diagnosis, prognosis

## Abstract

In recent years, it has become increasingly evident that cancer cells and the local microenvironment are crucial in the development and progression of tumors. One of the major components of the tumor microenvironment is the extracellular matrix (ECM), which comprises a complex mixture of components, including proteins, glycoproteins, proteoglycans, and polysaccharides. In addition to providing structural and biochemical support to tumor tissue, the ECM undergoes remodeling that alters the biochemical and mechanical properties of the tumor microenvironment and contributes to tumor progression and resistance to therapy. A novel concept has emerged, in which tumor-driven ECM remodeling affects the release of ECM components into peripheral blood, the levels of which are potential diagnostic or prognostic markers for tumors. This review discusses the most recent evidence on ECM remodeling-derived signals that are detectable in the bloodstream, as new early diagnostic and risk prediction tools for the most frequent solid cancers.

## 1. Introduction

Solid tumors are complex entities that are characterized by the coexistence of cancer cells and their microenvironment, composed of various cell types, including fibroblasts, adipocytes, endothelial cells, bone marrow-derived immune cells, and the extracellular matrix (ECM) [1]. All of these elements contribute to the development of cancer, which is not an entirely cancer cell-autonomous process but depends on the ability of cellular and noncellular components in the microenvironment to: i) establish a pro-tumor milieu, ii) regulate tumor cell behavior, and iii) coevolve with cancer cells [2,3]. This complex dynamic interaction between tumor cells and their microenvironment has been clearly demonstrated to influence the development, progression, and response to therapy of most solid cancers [4].

The ECM is a complex network of noncellular components, including structural proteins—predominantly collagens—, matricellular proteins—e.g., periostin, thrombospondins, osteopontin and secreted protein acidic and rich in cysteine (SPARC)—, glycoproteins, proteoglycans, and polysaccharides. These molecules contribute in deposition and arrangement of ECM and modulate cell-matrix interaction through their distinct biochemical and physical properties. In addition to its structural function, the ECM is highly dynamic and versatile and is an essential part of the tissue milieu, governing crucial aspects of cell biology. In this context, abnormal ECM dynamics contribute to cancer and lead to the conversion of the stem cell niche into a cancer stem cell niche, promoting the organization of premetastatic/metastatic environments and disrupting tissue polarity, inducing tissue invasion [5].

The composition and mechanical properties of the ECM modulate many of the tumor cell responses that represent the hallmarks of cancer, such as the evasion of apoptosis, insensitivity to growth inhibitors, sustained angiogenesis, self-sufficient growth, limitless replicative potential, and tissue invasion and metastasis [6]. The ECM is highly dynamic, versatile, and constantly remodeled as a result of activities of cells that reside within, which in turn are influenced by the ECM itself [5]. ECM remodeling is characterized by the increased synthesis and deposition of collagens and upregulation of matrix metalloproteinases (MMPs) [7]. These enzymes process matrix components, such as collagens [8], leading to the production and release of bioactive fragments mainly from non-fibrillar collagens [9]. MMP-derived changes in the tumor microenvironment typify tumor prognosis, supporting the function of structural remodeling of the ECM in the progression of many epithelial cancers, including breast, lung, and pancreatic tumors [10,11,12].

Consequently, interest in reactive stroma and ECM remodeling as potential tissue biomarkers in the management of cancer has grown. Cancer research in the past several decades has focused on identifying stroma/ECM-related characteristics as histological parameters [13] and gene-based signatures with diagnostic and prognostic significance in the most frequent solid tumors [14,15,16,17], with the possibility of being applied across several cancer types [18,19].

The tumor stroma is a potential source of new biomarkers, not merely in situ at the tissue level. ECM molecules that are derived from stromal changes are also released into the bloodstream and might represent surrogate markers of tumor development [3,20]. Thus, there is growing interest in studying ECM remodeling-derived molecules as circulating biomarkers. In this review, we discuss the results that have been obtained in the past 10 years of cancer research on the potential function of circulating ECM remodeling-derived molecules in the diagnosis and prognosis of solid tumors.

## 2. Presence of ECM Remodeling-Derived Molecules in Blood

Using a conditional transgenic HER2/neu-induced mouse model of breast cancer, Pitteri et al. [21] detected proteins that originated from the microenvironment in plasma, demonstrating that the plasma proteome is a sensitive biomonitor of tumor-host interactions. Applying a quantitative proteomic approach to plasma of these mice, the authors identified a set of proteins that changed in relative abundance during tumor induction/progression and regression upon doxycycline administration and withdrawal, respectively. Comparing plasma proteins detected in tumor-bearing mice to proteome, gene expression profiling and immunohistochemistry (IHC) of human breast cancer cell lines and murine induced breast tumors, circulating proteins resulted to originate from the tumor and local microenvironment. In plasma from mice with early-stage breast cancer, they observed higher levels of acute-phase response proteins, complement system proteins, and immune cell proteins, in addition to tumor microenvironment-specific molecules.

When considering the human context, although a direct demonstration of the stromal origin of circulating molecules is not achievable, the tumor stromal compartment was found positive for the same ECM remodeling-derived molecules that were detected in blood in various cancer types by immunohistochemical analysis of tumor tissues. Indeed, collagen 18, the precursor molecule of endostatin, which was upregulated in serum from colorectal cancer patients, was positive in basement membrane structures around invasive tumors and in desmoplastic stromal areas by IHC performed on tumor specimens of the same patients [22]. In non-small-cell lung cancer, higher serum levels of osteopontin, a glycophosphoprotein that has been implicated in tissue remodeling, were noted in patients with osteopontin-positive versus -negative lung cancers, as evaluated by IHC [23]. In breast cancer patients, IHC analysis on disease specimens showed stromal cell positivity of the same ECM molecules that, in blood, discriminated breast cancer patients from those with benign disease [24].

## 3. ECM Remodeling as Circulating Biomarkers in Cancer Diagnosis

The potential use of molecules that are derived from tumor-microenvironment crosstalk as circulating biomarkers has generated notable results, primarily in the diagnosis of cancer (Table 1). Early detection is a significant need in cancer management, because it influences patient mortality.

### 3.1. Breast Cancer

Although the use of standard screening tests in the management of breast cancer (BC), such as mammography, ultrasonography, and magnetic resonance imaging, have reduced patient mortality, early diagnosis with noninvasive procedures remains a challenge [50]. Several limitations that require invasive procedures persist, such as discriminating benign from malignant nodules in women with imaging that is merely suggestive of a tumor, which occurs in approximately 10% of women with a nodule detected during early screening.

Collagens are the main proteins of stromal origin that are detected in blood. In particular, collagen 4, as measured by enzyme immunoassay, is significantly higher in serum from patients with BC compared with healthy women [25]. MMP-degradation products of collagen 1 (C1M), collagen 3 (C3M) and collagen 4 (C4M and C4M12) are significantly elevated in serum from BC patients versus healthy donors, all with accuracies of over 75% in discriminating cancer patients [26].

Other ECM-derived molecules have been detected in plasma, such as collagen oligomeric matrix protein (COMP) and fibronectin. In particular, the combination of COMP, collagen 11, and collagen 10 has discriminatory capacity for BC patients versus healthy donors and patients with benign breast disease [24]. Plasma fibronectin levels are significantly higher in BC patients compared with healthy controls, patients with noncancerous diseases (e.g., inflammatory disease), and women with benign breast disease [27]. In addition, MMP-9 serum proteolytic activity is greater in women with malignant versus benign nodules [28].

By liquid chromatography–mass spectrometry (LC–MS/MS) analysis of plasma samples, Cohen and colleagues [29] demonstrated that a subset of peptides, corresponding to α-1-microglobulin/inter-α-trypsin inhibitor light chain precursor, gelsolin, clusterin, and biotinidase, classifies BC patients into two histological types: infiltrating ductal carcinomas and invasive mammary carcinomas with a lobular, tubular, mucinous, or medullary histotype. In addition, they found that circulating levels of a fibronectin-specific peptide differed significantly between triple-negative BC patients and patients who were positive for at least one receptor (estrogen, progesterone, or epidermal growth factor 2), indicating that these ECM-derived circulating biomarkers discriminate BCs of various subtypes during early detection.

### 3.2. Lung Cancer

For lung cancer, early diagnosis is also urgent, because the disease is often diagnosed at an advanced stage, for which there are no effective clinical options. Thus, the combination of diagnostic imaging by low-dose computed tomography (CT), which is the only evidence-based method for the early detection of lung cancer that reduces mortality, with new potential biomarkers is still a challenge [51]. In this context, many molecules that are derived from ECM remodeling have been analyzed with regard to their discriminatory potential in patients with lung cancer versus healthy controls.

Andriani and colleagues [30] reported that plasma samples from patients with lung cancer had significantly higher levels of collagen 10a1 and the collagen-binding matricellular protein SPARC compared with healthy controls who were matched for clinical parameters, such as sex, age, and smoking status. Levels of collagen 10 differed significantly only in females, whereas SPARC maintained significant differences between gender subgroups, with good overall discrimination of patients from controls.

Osteopontin, another protein that has been implicated in tissue remodeling, is significantly higher in serum from patients with lung cancer compared with healthy volunteers, and moreover, when smoking history was considered, the levels of circulating osteopontin were higher in smokers than in nonsmoking and ex-smoking lung cancer patients [31], suggesting that smoking status is an important parameter that must be taken into account when searching for new lung cancer biomarkers. ECM remodeling is an active process in the pathogenesis of lung cancer and chronic obstructive pulmonary disease (COPD) [52], rendering it difficult to identify microenvironment-derived markers that are specific for neoplastic conditions. Willumsen and colleagues [32] showed that MMP-degraded collagen 1 (C1M) and MMP-degraded citrullinated vimentin (VICM) are significantly elevated in serum from lung cancer patients compared with healthy donors, with excellent performance in detecting subjects with lung cancer. However, the diagnostic power of this two-marker combination fell dramatically when subjects with idiopathic pulmonary fibrosis or COPD were considered as the control group. In contrast, serum tumstatin, a matrikine protein that is derived from collagen 4a3, has tremendous diagnostic potential for non-small-cell lung cancer compared with healthy donors and patients with idiopathic pulmonary fibrosis or COPD as control groups [33].

### 3.3. Colorectal Cancer

Considering the gradual evolution of most cases of colorectal cancer (CRC) from adenomas, studies for the identification of diagnostic biomarkers in this pathology should also consider their ability to discriminate CRC from precancerous lesions [53]. MMPs and their tissue inhibitors are involved in tumor progression and the invasion of CRCs [54]. One of the most extensively studied MMPs, MMP9, is significantly elevated primarily in serum from CRC patients versus healthy controls [34,35,36,37,38]. Moreover, its enzymatic activity is improved in sera from patients with malignant tumors with respect to controls by gel zymography, reaching significant differences in stage II and III cancer. In contrast, the activity in serum from patients with adenomas remained consistently within the same range as in controls, suggesting the value of this biomarker in discriminating patients with adenomas from those with CRC [36].

Notably, a recent study by Gimeno-García and colleagues [38] analyzed plasma samples that were prospectively collected from patients who were undergoing colonoscopy and included nonadvanced adenomas, advanced adenomas, and CRCs. The levels of MMP9 were significantly higher in CRC patients compared with healthy controls and nonadvanced adenoma patients, whereas no significant differences were observed between CRC and advanced adenoma patients.

Another protein that discriminates between malignant and nonmalignant lesions is tissue inhibitor of metalloproteinase type 1 (TIMP-1). TIMP-1 levels are significantly higher in serum from CRC patients versus colorectal adenoma patients and controls [37], and a recent meta-analysis of 9 published studies, comprising 819 CRC patients and 1067 healthy controls, showed that circulating levels of TIMP-1 have significant clinical value with moderately high sensitivity and specificity in identifying CRC patients [55].

With regard to collagens, the plasma concentration of collagen 6a3 is significantly higher in CRC patients compared with healthy subjects [39], and serum levels of collagen 10 are significantly elevated in CRC and adenoma patients versus controls but fail to distinguish malignant and non-malignant lesions [40]. C1M, C3M, and collagen 3 (pro-C3) identifies CRC subjects with respect to controls, and C1M and pro-C3 are significantly higher in serum from CRC versus adenoma patients, rendering them potential markers in making an early differential diagnosis [7]. These biomarkers have also been considered in relation to the clinicopathological features of CRC patients—specifically, C1M, C3M, C4M, and pro-C3 are significantly higher in stage IV than in stages I, II, and III [7]. Similarly, serum endostatin levels are significantly lower in stage I patients compared with more advanced stages and significantly higher in T3-4 versus T1-2 patients, supporting the increase in remodeling of the tumor microenvironment during progression. Although it is significantly higher in serum from CRC patients compared with healthy controls, endostatin has not shown satisfactory discriminatory diagnostic power [22].

### 3.4. Urological Cancer

As in CRC, MMPs and their tissue inhibitors are widely considered to be circulating biomarkers in urological cancers, such as renal and bladder tumors. Two independent studies have demonstrated the value of this class of proteins. Serum levels of MMP2, MMP9, TIMP-1, and TIMP-2 are significantly lower in healthy controls compared with patients with metastatic renal cell carcinoma (RCC) [41]. MMP7, a promising tissue marker of poor prognosis [56], is significantly higher in the serum of node-positive and metastatic RCC patients than in those with localized RCC and in the serum of advanced versus nonadvanced stage patients [42].

In addition, this group reported the value of MMP7 in discriminating RCC patients from healthy controls, finding significantly lower levels in the latter. In urinary bladder cancer (UBC), serum MMP7 levels discriminate patients with metastases, wherein MMP7 is 2.9-fold higher in samples of patients with node-positive compared with node-negative tumors [43]. Serum levels of MMP9 and TIMP-2 are significantly greater in UBC patients versus age- and gender-matched healthy individuals [44].

### 3.5. Pancreatic Cancer

The stroma is the predominant constituent of pancreatic cancer (PC), forming approximately 80% of the tumor mass, especially in pancreatic ductal adenocarcinoma (PDAC), the most frequent pancreatic oncotype [20]. Therefore, based on the urgent clinical need for biomarkers in the early diagnosis of PDAC, stroma-derived molecules are garnering particular interest as circulating biomarkers. A panel of MMPs was analyzed in serum from PC patients—specifically, MMP-1, MMP-3, MMP-7, MMP-9, MMP-10, and MMP-12 were higher in cancer patients compared with healthy donors, showing good diagnostic accuracy, of which MMP-7 and MMP-12 achieved perfect discrimination [45].

Collagen proteins and their fragments are the most frequent biomarkers that are identified in the diagnosis of PC. A pilot study of control subjects and PC patients (*n* = 8 and 9, respectively) revealed elevations in circulating collagen 4 protein in serum samples from the latter [46]. A subsequent study by Willumsen and colleagues [20] confirmed this evidence in a larger number of samples. Specifically, they observed significantly higher levels of MMP-generated fragments of collagen 1, 3, and 4 in serum from PDAC patients compared with healthy controls, the combination of which attained extraordinary diagnostic power. Other soluble stroma-related molecules were found associated with PDAC, especially when considered in combination with tumor-related CA19.9 biomarker. Indeed Resovi and colleagues [47] demonstrated that both the combinations of CA19.9 with MMP-7 and with connective tissue growth factor (CCN2) display an almost perfect accuracy in discriminating PDAC patients from healthy subjects. Moreover, a panel consisting of CCN2, plasminogen (PGL), fibronectin, collagen 4 and CA19.9 was found able to distinguish PDAC from chronic pancreatitis patients [47]. The possibility of combining more than two biomarkers has also been considered by Franklin and colleagues [48], who evaluated four stroma-derived biomarkers—collagen 4, endostatin, tenascin C, and osteopontin—and 4 conventional markers—cancer antigens CA 19.9 and CA 125, CEA (carcinoembryonic antigen), and TPS (tissue polypeptide-specific antigen). Notably, in addition to the significantly higher levels of all stroma-derived proteins in PDAC patients versus controls, they found a narrower dynamic range of these markers in each group with respect to tumor-derived markers, strengthening their potential in the diagnostic setting. Collagen 6a3 is also differentially expressed at the tissue level between PDAC and adjacent nonmalignant tissue [57]. This protein is significantly higher in serum from PDAC patients compared with patients with benign lesions and healthy controls [49].

Overall, the existing evidence highlights the value of stroma-related circulating proteins as early diagnostic biomarkers, potentially overcoming the molecules that are strictly tumor-related.

## 4. ECM Remodeling as Circulating Biomarkers in Cancer Prognosis

In addition to early detection, an important priority is the identification of prognostic biomarkers that impact clinical decisions and overall outcomes. Based on the well-established involvement of ECM-derived molecules in the initiation and progression of cancer, they are also being examined as prognostic markers (Table 2).

### 4.1. Breast Cancer

MMP-mediated degradation products of collagens, studied extensively as diagnostic biomarkers in BC, were recently associated with patient outcomes—specifically, in two independent cohorts of metastatic BCs: hormone receptor-positive and HER2-positive. Levels of C1M, C3M, C4M, and pro-C3 were measured in serum from metastatic BC patients prior to initiation of therapy and were predictive of a shorter time to progression (TTP) and overall survival (OS) at high levels [58]. Specifically, in hormone receptor-positive tumors, higher levels of all collagen fragments were associated with significantly lower OS, and elevated C1M and C3M were linked to a shorter TTP. Similarly, in the HER2-positive metastatic BC cohort, elevated pro-C3 was significantly linked to shorter OS, whereas higher levels of all 4 molecules correlated significantly with shorter TTP.

The metastatic setting is the main context in BC that is considered with regard to determining the value of ECM-derived molecules as prognostic biomarkers. MMP2 serum levels are significantly higher in advanced HER2 positive BC patients who develop bone and central nervous system metastases compared with those without metastases [69]. Moreover, plasma levels of hyaluronic acid, in addition to being significantly higher in BC patients with versus without metastases, had independent prognostic value in the metastatic group. Considering the two independent metastatic BC cohorts, high levels of hyaluronic acid were significantly associated with shorter progression-free survival (PFS) and OS [59].

### 4.2. Lung Cancer

Osteopontin has been studied extensively as a circulating prognostic marker in lung cancer, alone and in combination with other molecules.

Two concomitant studies of nested patient samples, the Japan-Multinational Trial Organization (JMTO) Lung Cancer (LC) 0004 in the Japanese population and the SouthWest Oncology Group (SWOG) 0003 in the US population, obtained the same evidence regarding the prognostic significance of circulating osteopontin in advanced non-small-cell lung cancers [60,61]. In both studies, osteopontin was evaluated in samples from patients before treatment, and low levels were significantly associated with a favorable prognosis, in terms of OS and PFS; in the SWOG 0003 study, the prognostic value of circulating osteopontin was significant, regardless of treatment.

Consistent with these reports, the clinical value of circulating osteopontin was recently confirmed in the management of non-small-cell lung cancer [62] and in newly diagnosed primary lung cancer patients, in whom increased levels of osteopontin were significantly linked to worse survival [31]. Elevated osteopontin levels were significantly associated with shorter distant metastasis-free survival (DMFS) and OS; conversely, higher thrombospondin-1 content correlated significantly only with longer OS. The combination of the two proteins, expressed as the osteopontin:thrombospondin-1 ratio, was more significant than each individual molecule in primary non-small-cell lung cancer patients, wherein a rise in osteopontin:thrombospondin-1 ratio was associated with a 30% and 40% increased risk of metastasis and death, respectively [23].

### 4.3. Colorectal Cancer

In colorectal cancer, the prognostic value of circulating ECM-derived proteins is notable in such patients with metastases. Circulating levels of collagen 4 are significantly higher in CRC patients with versus without liver metastases. Moreover, in the former, these levels increase further at the time of disease progression compared with when metastasis is detected [63]. These data were validated in a second study from the same group in which they also demonstrated the prognostic significance of circulating collagen 4, in combination with carcinoembryonic antigen (CEA)—specifically, patients with CRC metastatic to the liver with low levels of both biomarkers experienced better OS compared with those with high levels of both markers (47% survival three years after surgery) [64].

The prognostic performance of circulating TIMP-1 has been examined extensively and was discussed in a meta-analysis of 10 studies that considered approximately 3000 CRC patients. High levels of TIMP-1 in plasma or serum were significantly associated with a poor prognosis in CRC patients, based on age-, gender-, grade-, and clinical stage-adjusted hazard ratios [65]. Bockelman and colleagues [66] measured MMP-8, MMP-9, and TIMP-1 in serum from CRC patients within 30 days prior to surgery and found that MMP-8 and TIMP-1 alone served as prognostic factors, high levels of which correlated significantly with worse five-year disease-specific survival (DSS). These proteins also had prognostic significance in subgroup analyses: high MMP-8 and TIMP-1 content was linked to poor DSS compared with low concentrations in left-sided CRC patients and patients with no systemic inflammation. Conversely, MMP-9, widely described as a diagnostic marker for CRC patients, reached significance as a predictor of DSS only when combined with TIMP-1 and expressed as the molar ratio MMP-9:TIMP-1.

### 4.4. Urological Cancer

In urological cancer, MMPs and their tissue inhibitors reflect the clinical course in such patients. In a cohort of metastatic clear cell RCC patients, MMP-9:TIMP-2 ratio was significantly elevated at the time of progression versus diagnosis and was a significant predictor of progression-free survival in univariate and multivariate analyses [41]. Further, Niedworok and colleagues [42] found that high levels of serum MMP-7 are strongly associated with an unfavorable prognosis, in terms of OS, DSS, and DMFS. Moreover, this biomarker is an independent prognostic factor of OS with greater significance than the presence of metastases and tumor grade and stage.

In urinary bladder cancer, circulating MMP-7 is a stage- and grade-independent prognostic factor of DSS and OS, wherein patients with high MMP-7 concentrations have a poor prognosis [67]. In addition, endostatin, the generation of which is strictly related to MMP-7, is a prognostic marker—high serum levels correlate significantly with unfavorable DSS and DMFS in bladder cancer patients [68].

### 4.5. Pancreatic Cancer

In contrast to the other solid cancer types that we have discussed, ECM-derived circulating molecules have been poorly studied as prognostic biomarkers in PC. A study of a small tumor cohort (*n* = 14) reported that plasma levels of collagen 4, measured after surgery, in addition to having diagnostic value, correlated with patient prognosis; specifically, PC patients with high levels of collagen 4 experienced shorter survival than those with low levels [46]. Similarly, Franklin and colleagues [48] showed that in the postoperative setting, only stroma-derived circulating markers, such as collagen 4, endostatin, and osteopontin, were associated with shorter survival at high levels, whereas conventional tumor markers, such as CA19.9, CA125, and CEA, failed.

Overall, independent of the ECM-derived molecule and cancer type, higher levels of this class of molecules are generally associated with an unfavorable prognosis in cancer patients.

## 5. Conclusions

Circulating biomarkers in cancer remain a challenging research topic—the possibility of detecting incipient tumor cells significantly increases the potential for treatment and a cure. In contrast to invasive procedures, such as biopsies, which cannot be performed repeatedly and are at times even impractical, tumor-derived molecules in the blood have the advantage of being able to be measured using minimally invasive methods that allow frequent repeat testing during patient follow-up. Thus, in cancer, noninvasive modalities for early detection and risk assessment should become a priority.

Several serum-based tumor markers that are derived solely from neoplastic cells have been described, but none are used for tumor diagnosis—merely to monitor treatment response. Thus, new strategies are continually being developed, one of which is represented by molecules that are derived from the interplay between a tumor and its microenvironment. Such crosstalk fosters and amplifies stromal changes that ultimately manifest in the bloodstream.

Because tissue remodeling commonly occurs in cancers, microenvironment-related molecules might be useful in detecting and monitoring tumors of many oncotypes, as demonstrated for collagen degradation products and MMPs, which are prevalent in the bloodstream of patients with various tumors.

In this context, the C-terminal portion of collagen 8 is increased in the serum of patients who have been diagnosed with breast, lung, prostate, colon, and ovarian carcinoma and melanoma, compared with healthy subjects. MMP-degraded collagen 1 (C1M) and MMP-degraded vimentin (VICM), are significantly higher in serum from lung, gastric, prostate, and melanoma cancer patients versus controls. Similarly, collagen 4 and MMPs and their tissue inhibitors have prognostic value in various solid cancers, such as CRC, urological cancers, and pancreatic cancer. The ability of circulating collagen 1 and 4 to reflect the presence of a tumor is likely attributed to the abundance of collagen 1 in the stroma, where tumors develop, and the finding that collagen 4 is a core component of all basement membranes including those at the tumor and vasculature level in nearly all solid cancers.

Other stroma-derived molecules have been found to be specific for certain cancer oncotypes, such as MMP7 and osteopontin for urological and lung cancer, respectively. Thus, combining ECM molecules could constitute a new tool for detecting incipient tumor cells and, in some cases, determining their origin. A multimarker test based on stroma-related circulating molecules, combined with biomarkers that are related to the characteristics of tumor cells (e.g., CA-125, CEA, CA 15-3 CA19.9, TPS), is a promising platform for tumor diagnosis and to perform patients’ follow-up through blood analysis.

The detection of tumor-stroma interplay in the blood might be a feasible strategy for overcoming intratumor heterogeneity, which impedes diagnostic approaches that are based on soluble tumor-related molecules. ECM-related molecules that are detectable in the bloodstream sense a tumor independently of the complexity of its clonal evolution, as supported by the results of cohorts of metastatic cancer patients in whom circulating ECM-related markers were predictive of TTP and OS. This finding suggests that remodeling of the tumor microenvironment also occurs during metastatic dissemination at various sites and that this phenomenon mainly involves the same molecules that are implicated in the development of tumors at primary sites.

In conclusion, these studies provide proof of concept that tissue remodeling is a promising source of novel biomarkers in the management of cancer. Searching for novel tumor biomarkers in circulation beyond the cancer cell itself is a new noninvasive diagnostic and prognostic approach, if we consider that these biomarkers are easily detectable by enzyme-linked immunosorbent assay, alone or combined in multiplex assays.

The next step in this endeavor is to validate the clinical value of circulating stroma-derived molecules by designing large prospective studies in which their diagnostic and prognostic capacity is analyzed with regard to the presence and progression of tumor cells and the existence of non-neoplastic alterations, such as fibrosis and local inflammation that could occur before development of a tumor.

Since molecules deriving from tumor-stroma interplay potentially overcome intra-tumor heterogeneity and are shared by multiple tumor oncotypes, their targeting even more opens the possibility of cancer therapeutic purposes. In this context, strategies aimed at targeting molecules involved in ECM remodeling such as MMP, transforming growth factor-β, enzymes involved in collagen cross-linking (lysyl oxidase and lysyl oxidase-like 2) are being considered as potentially new therapeutic tools [70]. Clinical trials are currently ongoing to demonstrate the efficacy of these approaches in inhibiting cancer progression [71].

## Figures and Tables

**Table 1:** Extracellular matrix (ECM)-derived molecules in cancer diagnosis.

Solid Cancer Type	ECM Molecules	Diagnostic Setting	Author (Reference)
Breast cancer (BC)	COL4	BC patients vs. healthy donors	Mazuoni [25]
	C1M, C3M, C4M, and C4M12	BC patients vs. healthy donors	Bager [26]
	COMP, COL11, and COL10	BC patients vs. healthy donors	Giussani [24]
		BC vs. benign breast disease	
	Fibronectin	BC patients vs. healthy donors	Moon [27]
		BC vs. benign breast disease	
	MMP-9	BC vs. benign breast disease	Golubnitschaja [28]
	α-1-microglobulin/inter-α-trypsin inhibitor light chain precursor, gelsolin, clusterin, and biotinidase	Infiltrating ductal BC vs. other infiltrating histotype	Cohen [29]
	Fibronectin	Triple-negative vs. hormone-positive BC	Cohen [29]
Lung cancer (LC)	COL10 and SPARC	LC patients vs. healthy donors	Andriani [30]
	Osteopontin	LC patients vs. healthy donors	Kerenidi [31]
	C1M, VCIM	LC patients vs. healthy donors	Willumsen [32]
	Tumstatin (COL18A1)	LC patients vs. healthy donors	Nielsen [33]
		LC vs. COPD ^1^ or idiopathic pulmonary fibrosis	
Colorectal cancer (CRC)	MMP-9	CRC patients vs. healthy donors	Wilson [34]; Emara [35]; Biasi [36]; Mroczko [37]
	MMP-9	CRC patients vs. healthy donors	Gimeno-Garcia [38]
		CRC vs. nonadvanced adenomas	
	MMP-9 activity	CRC vs. adenomas	Baisi [36]
	TIMP-1	CRC patients vs. healthy donors	Mroczko [37]
		CRC vs. adenomas	
	COL6A3	CRC patients vs. healthy donors	Qiao [39]
	COL10	CRC patients vs. healthy donors	Solé [40]
		Adenoma patients vs. healthy donors	
	C1M, C3M, and pro-C3	CRC patients vs. healthy donors	Kehlet [7]
	C1M and pro-C3	CRC vs. adenomas	Kehlet [7]
	C1M, C3M, C4M, and pro-C3	Stage IV vs. stage I-II-III CRC	Kehlet [7]
	Endostatin (COL18A1)	Stage I vs. advanced stage CRC	Kantola [22]
		Advanced stage (T3–4) vs. nonadvanced stage (T1–2)	
Urological cancer	MMP2, MMP9, TIMP-1, and TIMP-2	Metastatic RCC patients vs. healthy donors	Miyake [41]
(Renal cell carcinoma—RCC; Urinary bladder cancer—UBC)	MMP7 MMP7	Metastatic or node-positive RCC vs. localized RCC Advanced stage vs. nonadvanced stage RCC Node-positive vs. node-negative UBC patients	Niedworok [42] Szarvas [43]
	MMP9 and TIMP-2	UBC patients vs. healthy donors	Ramón de Fata [44]
Pancreatic cancer (PC)	MMP1, MMP3, MMP7, MMP9, MMP10, and MMP12	PC patients vs. healthy donors	Kahlert [45]
	COL4	PC patients vs. healthy donors	Ohlund [46]
	C1M, C3M and C4M	PDAC ^2^ vs. healthy donors	Willumsen [20]
	MMP7 and connective tissue growth factor	PDAC ^2^ vs. healthy donors	Resovi [47]
	MMP7, connective tissue growth factor, plasminogen, fibronectin and COL4	PDAC ^2^ vs. chronic pancreatitis patients	
	COL4, endostatin (COL18A1), tenascin C, and osteopontin	PDAC patients vs. healthy donors	Franklin [48]
	COL6A3	PDAC patients vs. healthy donors PDAC vs. benign lesions	Kang [49]

COPD: chronic obstructive pulmonary disease; ^2^ PDAC: pancreatic ductal adenocarcinoma.

**Table 2:** ECM-derived molecules in cancer prognosis.

Solid Cancer Type	ECM Molecules	Patients	Prognostic Setting	Author (Reference)
Breast cancer (BC)	C1M, C3M, C4M, and pro-C3 C1M and C3M pro-C3 C1M, C3M, C4M, and pro-C3	Hormone receptor + metastatic BC HER2 + metastatic BC	shorter OS ^1^ shorter TTP ^2^ shorter OS shorter TTP	Lipton [58]
	hyaluronic acid	Metastatic BC	shorter PFS ^3^ shorter OS	Peng [59]
Lung cancer (LC)	osteopontin	advanced non-small-cell lung cancer	shorter OS and PFS	Isa [60]; Mack [61]
	osteopontin	non-small-cell lung cancer	worse OS	Takenaka [62]
	osteopontin	primary lung cancer	worse OS	Kerenidi [31]
	osteopontin	primary non-small-cell lung cancer	shorter DMFS ^4^ and OS	Rouanne [23]
	Thrombospondin-1		longer OS	
	osteopontin/thrombospondin-1		↑ risk of metastases and death	
Colorectal cancer (CRC)	COL4	Metastatic CRC	at time of disease progression	Nystrom [63]
	COL4 and CEA	Liver metastatic CRC	worse OS	Nystrom [64]
	TIMP-1	CRC	poor prognosis	Lee [65]
	MMP8	CRC left-sided CRC and no systemic inflammatory condition	worse DSS ^5^ worse DSS worse DSS	Bockelman [66]
	TIMP-1	CRC left-sided CRC and no systemic inflammatory condition	worse DSS worse DSS worse DSS	
	MMP9/TIMP-1 ratio	CRC	longer DSS	
Urological cancer	MMP9/TIMP-2 ratio	Metastatic clear-cell RCC	shorter PFS	Miyake [41]
(Renal cell carcinoma—RCC; Urinary bladder cancer—UBC)	MMP7 MMP7	RCC UBC	poor OS, DSS, and MFS ^6^ poor DSS and OS	Niedworok [42] Szarvas [67]
	endostatin	UBC	poor DSS and MFS	Szarvas [68]
Pancreatic cancer (PC)	COL4	PC	shorter OS	Ohlund [46]
	COL4, endostatin (COL4A3), and osteopontin	PC	shorter OS	Franklin [48]

^1^ OS: overall survival; ^2^ TTP: time to progression; ^3^ PFS: progression-free survival; ^4^ DMFS: distant metastasis-free survival; ^5^ DSS: disease-specific survival; ^6^ MFS: metastasis-free survival.
